# Supplementary material for: Investigation of Genetic Variants Associated with Tryptophan Metabolite Levels via Serotonin and Kynurenine Pathways in Patients with Bipolar Disorder
Source: Metabolites. 2022 Nov 17;12(11):1127. doi: 10.3390/metabo12111127 (PMC9694761; doi:10.3390/metabo12111127)
Supplement: Supplementary file 1 [file metabolites-12-01127-s001.zip › metabolites-1996139-supplementary.pdf]

Table S1. SNPs located in the *IDO1* and *IDO2* genes found to be nominally associated with the KYN/TRP ratio

CHR	SNP	EA	OA	BETA	P
IDO1					
8	rs6474193	T	C	6.83	0.0014
8	rs7820268	T	C	6.38	0.0028
8	rs10108662	A	C	6.15	0.0033
8	rs7010461	T	C	6.38	0.0028
IDO2					
8	rs117615420	A	C	8.89	0.034
8	rs2340953	C	T	6.61	0.048
8	rs1971708	T	C	-6.50	0.021
8	rs113833094	C	T	8.65	0.026
8	rs116929069	A	G	8.65	0.026
8	rs113043725	T	C	8.65	0.026
8	rs73621342	A	G	8.65	0.026
8	rs112487431	T	G	8.65	0.026
8	rs147162253	G	A	-10.24	0.021
8	rs73605122	G	A	8.65	0.026

Analyses were adjusted for sex and diagnosis (patient with BD or control). The effect of diagnosis was not significant for any SNP.

Abbreviations: CHR, chromosome; EA, effect allele; OA, other allele; SNP, single nucleotide polymorphism

Table S2. SNPs located in the *KMO* gene found to be nominally associated with the 3-HK/KYN ratio

CHR	SNP	EA	OA	BETA	P
1	rs6658805	G	C	-16.19	0.039
1	rs12138459	A	G	21.11	0.014
1	rs3765806	G	C	20.58	0.015
1	rs12118651	T	C	20.58	0.015
1	rs10926517	A	T	20.58	0.015
1	rs12139931	G	T	20.58	0.015
1	rs10802971	G	C	20.58	0.015
1	rs3602627	A	G	-18.26	0.019
1	rs6429281	T	C	23.03	0.020
1	rs3765809	T	G	23.44	0.019

Analyses were adjusted for sex and diagnosis (patient with BD or control). The effect of diagnosis was not significant for any SNP.

Abbreviations: CHR, chromosome; EA, effect allele; OA, other allele; SNP, single nucleotide polymorphism

Table S3. SNPs located in the *KYNU* gene found to be nominally associated with 3-HK levels

CHR	SNP	EA	OA	BETA	P
2	rs11903769	A	G	-3.78	0.044
2	rs11903737	T	C	-3.78	0.044
2	rs7424573	T	C	-3.78	0.044
2	rs7600006	T	C	-2.90	0.045
2	rs6756023	A	G	-2.90	0.045
2	rs12473916	G	A	-2.90	0.045
2	rs12473966	G	A	-2.90	0.045
2	rs4662307	C	T	-2.90	0.045
2	rs12475145	G	A	-2.90	0.045
2	rs6739188	T	A	-2.90	0.045
2	rs35202501	G	C	-3.78	0.044
2	rs2083373	G	C	-2.90	0.045
2	rs2083374	C	T	-2.90	0.045
2	rs6429992	C	T	-2.90	0.045
2	rs6429993	C	T	-2.90	0.045
2	rs2381355	C	A	-2.90	0.045
2	rs13393769	T	C	-3.78	0.044
2	rs6734299	C	A	-2.90	0.045
2	rs6709193	T	C	-2.90	0.045
2	rs6713123	A	G	-2.90	0.045
2	rs164732	T	C	-2.90	0.045
2	rs351707	A	T	-2.90	0.045
2	rs351706	T	G	-2.90	0.045
2	rs11692509	C	T	-3.78	0.044
2	rs12474744	A	G	-3.78	0.044
2	rs463247	G	C	-2.90	0.045
2	rs351701	G	C	-2.90	0.045
2	rs11692287	A	G	-3.78	0.044
2	rs3768851	A	C	-3.78	0.044
2	rs3768850	T	C	-3.78	0.044
2	rs164731	T	A	-2.90	0.045
2	rs6755074	G	T	-3.78	0.044
2	rs351708	G	A	-2.90	0.045
2	rs352893	A	C	-3.43	0.020
2	rs10179354	A	G	-4.23	0.032
2	rs13030111	A	G	-5.16	0.014
2	rs62169869	T	C	-5.16	0.014
2	rs3768848	A	G	-5.16	0.014
2	rs17805827	A	T	-5.16	0.014
2	rs3768847	G	A	-5.16	0.014
2	rs1388974	T	C	-5.16	0.014
2	rs12990543	G	A	-5.16	0.014
2	rs34082230	T	A	-5.16	0.014
2	rs13019625	G	A	-3.72	0.032
2	rs62169913	A	G	-3.82	0.028

2	rs11686073	A	G	-3.72	0.032
2	rs6429997	G	A	-3.13	0.038
2	rs62169914	G	A	-3.82	0.028
2	rs6734849	G	T	-3.13	0.038
2	rs960822	G	A	-3.13	0.038
2	rs1371515	G	T	-3.28	0.017
2	rs352874	T	G	-3.03	0.029
2	rs11902056	A	G	-3.27	0.033
2	rs352892	A	C	-3.41	0.029
2	rs352889	A	G	-3.41	0.029
2	rs62169918	C	T	-4.06	0.021

Analyses were adjusted for sex and diagnosis (patient with BD or control). The effect of diagnosis was not significant for any SNP.

Abbreviations: CHR, chromosome; EA, effect allele; OA, other allele; SNP, single nucleotide polymorphism
